# Surgery of War

**Published:** 1917-11

**Authors:** 


### SURGICAL

**Surgery of War.** Conclusions Adopted by the Interallied Surgical Conference (England, Belgium, France, Italy, Japan, Portugal and Serbia[1]) relative to the Treatment of War Wounds. Trans. from the *Archives de Médecine et de Pharmacie Militaires*, April 1917.

### I. — General principles in the treatment of war injuries, especially with regard to the prophylaxis of infection and to the sterilization of wounds.

1. The organization of the services should permit of the continuity of surgical direction in the treatment of the wounded.

2. At the battle posts and especially in the trenches, surgical aid ought to be reduced to a minimum. It should be confined to warding off complications which might prove immediately fatal and to the protection of wounds from infection. The wound should not be explored or washed : it should simply be covered with a dry dressing, either aseptic or antiseptic.

3. The wounded should be transferred as quickly as possible to one of the large hospitals situated along the front at a distance of from 10 to 20 kilometers from the lines.

4. It is well for each of these hospitals to have one or more annexes nearer to the firing line to receive as early as possible the more serious cases (shock, serious hemorrhage, wounds of the thorax or abdomen, etc.).

5. In general, war wounds should be considered as contaminated or infected.

6. The ends to be attained by treatment are :

*a*. Prevention of infection if the wound is only contaminated, or sterilization, if infection has set in.

*b*. The possibility of suture when clinical sterilization has been effected.

7. Wide opening of the wound and resection of the injured tissues and removal of bits of clothing or other foreign bodies,

1. The delegates from the United States and Russia could not attend the two first sessions.

should be regarded as a regular practice, except in benign cases which may be closely watched.

8. After such treatment immediate suture may lead to good results, especially in wounds of the joints. It should be performed only for recent wounds (at most not over eignt hours old) and when the surgeon can watch the patient for a period of fifteen days.

9. If immediate suture has not been performed, recourse may be had to secondary suture as soon as sterilization of the wound has been clinically recognized to be sufficient.

10. The progress of the wound ought to be systematically controlled by successive bacteriological tests in series, so as to ascertain the bacterial curve and the degree of sterilization.

11. In the case of hurried removal of patients whose wounds have been opened and excised, it is well to apply a dressing the action of which would continue during the period of transportation. There is room for investigation along this line.

12. There are several methods of progressive sterilization of wounds which allow regularly of secondary suture.

### II. — Study of laboratory methods for the purpose of aiding the surgeon in the choice of treatments.

1. In every important surgical service there should be an experienced bacteriologist with sufficient competent assistants and a laboratory adequately equipped.

2. The treatment of war wounds calls for close cooperation of surgeon and bacteriologist. The bacteriologist should himself come in direct contact with the wounded and should study and consult with the surgeon as to the investigations to be made and the treatment to be applied.

3. In general the investigations should be bacteriological, cytological, and humoral.

4. There is no reason to confine investigations within narrow limits; so far as indications are concerned, however, the principle problems for research appear to be the following:

*a.* The bacteriological flora of the wound at the start.

*b.* The bacteriological flora of the wound before and after surgical treatment, or before and after any considerable transfer of the patient.

*c.* The bacteriological flora of wounds which continue to suppurate or which present a complication.

*d.* Bacteriological control followed by sterilization of the wounds with a view to suture.

*e*. Bacteriological and biological control of the wounds for the purpose of determining the efficacity of various methods of treatment.

*f*. Examination of the blood for bacteria, the number of formed elements, and defensive properties; also for possible indications for the transfusion of the blood.

*g*. Special infections of certain anatomic regions, such as, articulations, serous membranes, mucous membranes, muscles, brain tissue, and cerebro-spinal fluid. Bacteriological, cytological, and humoral studies should be made along these lines.

*h*. The general indication for, and the application of, vaccine therapy.

5. In addition to laboratories, attached to the services, for the purpose of *practical* surgical assistance, more *laboratories should be established for scientific research along general lines* with regard to war surgery; and those already in operation should be adapted and organized more specifically for this purpose.

### III. — Comparative study of the different methods of extracting projectiles.

1. A thorough radiological examination should be made of each case as soon as general conditions permit.

2. The extraction of projectiles ought not to be attempted without some method of localization.

3. In the surgical services at the front, especially at times of great activity, radioscopic methods are most desirable.

4. The surgeon should without fail have at his disposal during an operation some one of the numerous apparati of direction and control (radiograph, stereoscope, electro-vibrator, compass, finger-stall telephone, or intermittent controller of rays).

5. In fixed services during periods of moderate activity, radiographic methods are sufficiently indicated.

6. The Conference calls especial attention to the necessity of providing every radiographic apparatus with all possible means of protecting the surgeon and radio-operator from the X-rays.

### IV. — Gaseous gangrene : its pathological and bacteriological aspects.

A. *Pathological.* — Under this heading will be considered the so-called " favorable " conditions.

At present, especially from a surgical point of view, predisposing conditions play the most important rôle, because at least some of them are amenable to therapeutic treatment.

Predisposing causes may be classed as general and individual.

a. *Predisposing causes of a general order.*

1. Geographic variations : the frequency and the clinical type of gaseous gangrene seem in a measure to be influenced by geographical environment.

2. Wounds from artillery projectiles or grenade explosions are the most dangerous.

b. *Predisposing causes of an individual order.* — Certain characteristics are almost constantly found in wounds which tend towards gangrene; the most important are :

1. The situation of a wound in the fleshy parts of a limb, especially of a lower limb.

2. The special character of a wound, such as a narrow orifice with deep and important lesions.

3. The presence of foreign bodies, and especially of bits of clothing, which may have on them earth with its infectious or fecal matter.

4. Extended contusion of the tissues, with partial devitalization.

Other predisposing conditions, less constant but frequent, are :

5. Great destruction of the bone or of an articulation.

6. Ischemia resulting from vascular lesions, from ligatures, and from a prolonged application of a tourniquet.

7. Multiplicity of wounds.

8. Hematoma.

9. Genuine traumatic shock.

10. Chances of gangrene are reduced according to the promptness of adequate surgical treatment.

11. Even after the proper treatment, gaseous gangrene may result from the too hasty removal of a patient, or from an insufficient method of treatment, especially after the application of dressings which permit the desiccation of wounds.

12. Attention is especially called to certain causes of intoxication in gaseous gangrene, and more particularly to acidosis whether general or local. Therapeutic indications may result.

B. *Bacteriological.* — 1. Investigations carried on from the beginning of the war have confirmed the infectious origin of gaseous gangrene and the anaerobic character of the bacteria which provoke it.

2. Among the bacteria encountred are to be noted the vibrion septic (Bacillus of malignant œdema), the Bacillus bellonensis (the precise relationship between the Bacillus bellonensis and the

Bacillus œdematiens is still undetermined), and the Bacillus perfringens (aerogenes capsulatus).

3. Infection is often aggravated by the presence of additional bacteria, especially the streptococcus.

4. There is much need of research for the purpose of obtaining active serums against the above-mentioned bacteria.

Such sera have been prepared, and their activity on animals has been proved.

### V. — Treatment of gaseous gangrene.

1. Gaseous gangrene is very often the result of delayed or insufficient surgical treatment.

2. Prophylactic treatment is most important : this necessitates the early removal of the wounded to surgical centers, where they may receive adequate care; complete opening of the wounds; excision of devitalized or gangrenous tissues; removal of all foreign substances; and careful hemostasis.

3. In the localized varieties with or without infiltration, curative treatment consists in :

*a.* Complete exposure to the air of deep pockets.

*b.* The excision of all gangrenous or suspected tissues.

*c.* The practice of lengthy incision into the region of infiltration and to the limits of this region.

4. *Amputation* is imperative in the case of *massive* gangrene, especially if it is complicated with bony or serious vascular lesions, and of gangrene of a *rapidly spreading* variety, accompanied by a generally alarming condition. (A quick fall in blood pressure is, in this connection, an important symptom.) The operation generally chosen is a flapless amputation.

When the amputation is to be made in healthy tissue, our English colleagues recommend an operation employing short flaps held apart. When the amputation is to be made in an infiltrated region, it is best to open wide the infiltrated part of the stump by incisions reaching to the upper limit of the member or to the trunk[1].

5. *Anesthesia* by means of nitrous oxide with oxygen is considered the best; when this is not to be had, ether may be substituted.

6. When projectiles are to be extracted, or *secondary* and *late*

1. MM. Depage and Derache add that interstitial injections of oxygen, superficial or deep, made above and below the wound as far as the distention of the skin, is a valuable adjuvant in surgical treatment.

operations are to be performed, it must be remembered that the process may give rise to gangrene.

### VI. — Traumatic shock.

Traumatic shock is a disturbance in the functional equilibrium, characterized clinically by reactions of a depressive nature which may result in death. The essential phenomena are cooling of the body and low blood pressure.

It is often accompanied by serious hemorrhages or acute toxemia which make it especially difficult to diagnose.

It may be caused or aggravated by pain, cold, and fatigue.

Diastolic blood pressure continuing at less than 60 mm. (Pachon), independent of hemorrhage or toxemia, is an indication for treatment to be applied *manometer in hand.*

This treatment may be summarized as follows :

*a. At regimental posts and divisional rescue stations :*

1. Injection of stimulants, especially camphorated oil (dose 10 cc.) repeated every two hours, is often the only treatment upon which the surgeon may rely.

2. The wounded should be removed when possible in heated ambulances.

*b. At advanced services or hospitals at the front :*

The posture given to the wounded, the heating of the body, and the reestablisment of normal blood pressure, constitute the three essentials in any treatment.

1. The patient should be placed in a horizontal position or one slightly inclined. This position is always to be avoided in wounds of the chest or of the brain. A compressive rolled bandage may be applied to the limbs.

2. The surface of the body should be kept carefully in an atmosphere heated to about 40°C. The restoration of body heat is an important indication in the treatment.

3. Absolute repose is imperative. Every useless movement and every cause of pain and excitement must be avoided.

4. Enteroclysis (drop method) of an isotonic solution with 1 o/o alcohol added, is advisable.

5. An attempt should be made by means of intravenous injections of saline solutions to reestablish the blood pressure. These injections may be made in large or small repeated doses.

Small *repeated* doses (250 cc. maximum) are preferable in genuine cases of shock without an important loss of blood. The effect upon the blood pressure is more lasting when the injection is made at the end of the operation. Later injections should be administered in accordance with the curve for the blood pressure, which should be determined by half-hourly observations. A new injection should be made every time the curve assumes an unfavorable bend[1].

6. *Is shock an indication or a counter-indication for operation?* The answer to this question depends upon the seriousness of the shock and the *nature* of the operation.

If the shock itself — aside from the hemorrhage — is serious, if the patient is cold and without a pulse, the shock must be treated first. The same is true if the operation to be performed is long or complex, as in the case of abdominal operations. Any considerable injury to the members calling for amputation is, nevertheless, an indication for operation.

Local anesthesia combined with general anesthesia by means of nitrous oxide is the best. Next to this ether appears to be the least harmful. Spinal injections have produced varying results according to the surgeons employing them, especially in amputations of the lower limbs. The use of chloroform is dangerous.

7. A condition of shock calls for the quickest possible process of operation, — the simpler the better, — with complete hemostasis (preventive, temporary, and permanent).

### VII. — Amputations.

1. Amputation is desirable only when the preservation o the limb would result in death or when its loss is certain.

1. As an isotonic solution to be injected the Locke solution is preferable.

| | |
|---|---|
| Chloride of Sodium. . . . . . . . . . . . . . . . . . . | 8.0 gm. |
| Chloride of Potassium. . . . . . . . . . . . . . . . . | 0.2 — |
| Bicarbonate of Soda. . . . . . . . . . . . . . . . . . | 0.2 — |
| Chloride of Calcium. . . . . . . . . . . . . . . . . . | 0.1 — |
| Glucose. . . . . . . . . . . . . . . . . . . . . . . . | 1. — |
| Water. . . . . . . . . . . . . . . . . . . . . . . . . | 1000. — |

The injection should be made slowly, lasting about ten minutes. If a second injection is necessary, a solution with a larger proportion of calcium than Locke's solution (hypertonic solution) may be employed.

If the blood pressure falls a great deal, a third injection should contain adrenalin (added at the time of using). Just as the injection is to be made the following ingredients may be mixed : 0.5 cc. of the solution 1 to 1000 adrenalin; and 50 cc. of isotonic solution of the first formula. The injection should always be made slowly, never in less than 10 minutes. Only completely colorless adrenalin should be used. The action of the adrenalin and of the pituitary extract is short-lived. The transfusion of blood seems also to give results as in the case of hemorrhage.

2. The two chief indications for amputation are extensive traumatism and infections.

3. Amputations for complicating infections are always very serious. Statistics based upon 29,139 amputations, show a mortality of 28 o/o in cases of infection against the usual mortality of 6 o/o. Statistics drawn from 3,633 disarticulations lead to the same conclusions.

4. Amputation is called for less frequently in injuries to the *upper limbs* than is the case with the lower limbs. Less serious and less frequent infection, the favorable results of resection, and the unsatisfactory nature of substitutes for the upper limbs, are the chief reasons for this difference.

5. Indication for *primary* amputation is extensive traumatism; such as, laceration, crushing, and incomplete separation of the limb, especially if there is a rupture of the great veins and arteries.

6. An indication for *secondary* amputation is dry or seriously infectious gangrene. Other ascending infections (superficial gangrene) are amenable to conservative treatment.

7. *Late* amputations are indicated in chronic infections by cachexia, which is not amenable to treatment.

*Primary* amputation or that within 24 to 48 hours, should be effected as nearly in the region of the fracture as the seriousness of the lesions will permit. The operation should be by simple section of the soft parts or with rectification of the bones.

Amputation for *infection* should be *flapless* or with short flaps held apart. If there is need, it can be *revised* when the wound has been disinfected and the soft parts have been given all the elongation possible.

The technique of a *delayed* amputation should be determined largely by the requirements of an artificial limb.

In cases of fracture complicated by extended fissures of the bone, amputation may be performed in the region of the wound, thus preserving the fissured bone, provided sufficient disinfection of the wound is possible.

1. In the case of serious shock, the use of nitrous oxide and oxygen is desirable; ether is the next best anesthetic.

2. In general, except in extreme cases, shock is not a counter-indication for amputation.

3. Preventive hemostasis is necessary: it should be complete.

4. The periostium should be neatly cut without causing separation.

5. In primary amputations, especially for infection, the wound ought to be left wide open. The operator should be on his guard against interstitial separations. English surgeons even advise leaving the blood vessels ligatured and the nerves exposed at the surface of the wound.

6. After the operation, the chief precautions should be : 1. The disinfection of the wound against osteomyelitis; 2. The control of the healing of the flaps.

7. More attention should be paid to the length of the stump than to the amount of remaining flesh.

*Amputations of the lower limbs* :

The disarticulation of the hip even by the raquet process with preventative ligature of the vessels, is more serious than an inter-trochanteric amputation.

A ***thigh*** stump ought to be at least 12 to 14 cm. in length below the great trochanter in order to permit of a successful artificial limb.

The amputation of the thigh in the *upper region* makes it difficult to provide a substitute because of the removal of the femur.

An amputation in the *middle* or *lower* region gives good results. The sciatic nerve must be cut above the flap.

The *Gritti* operation should not be praticed except in delayed amputation.

The disarticulation of the *knee* is effective as a preliminary operation.

Amputation in the *plateau of the tibia* gives good results, as it permits walking with the stump of the knee.

Amputation of the *leg* should be as low as possible; the posterior flap is preferable; the fibula should be cut about an inch above the tibia.

Excellent results may also be obtained by *tibio-tarsal disarticulation* with section of the malleoli, *sub-astragalus disarticulation*, *Lisfranc*, *osteoplastic amputations*, Syme, Pyrogoff.

The *Chopart* operation may be practiced in secondary and delayed amputation.

Any resection of the bones of the foot which leaves a good plantar sole with normal axis of the leg, should be preferred to amputation.

*Amputations of the upper limbs :*

In amputations at the *shoulder* it is well to preserve the head of the humerus.

Amputations of the *arm* should be as low as possible — 10 cm. of bone are needed for a stump. The method may be either circular or with flaps.

In amputations of the *fore-arm* leave, whenever possible, 10 cm.

of bone below the elbow for leverage. The movements of pronation and supination should be maintained.

On the *hand* only primary rectifications should be made, unless more complete operations are necessary, because of the service value of every segment preserved.

Treatment of joints above the place of amputation as well as the exercise of the muscles, should be continued during the entire period of healing.

The artificial limb should be applied as soon as possible. The use of a temporary apparatus is often called for, especially in the case of the lower limbs.

The " *cinematic* " method may afford indications in the case of the arm and fore-arm.

### VIII. — Artificial Limbs.

1. Artificial members should be functional and esthetic; that is, they should, if possible, restore the function and the form of the amputated part. They should be supplemented by a functional and professional reeducation.

2. Substitution ought to be as early as possible. In the case of lower limbs it should be made as soon as the condition of the wound permits. The use of crutches should be limited as far as possible. To enable the patient to walk before his wound is healed, temporary supports may be devised, the best being those contrived by the surgeon himself.

3. *For the upper members* the substitutes are made of leather with metal splints. They include :

a. *An assistance arm*, a kind of simple, cheap substitute which serves a laborer fairly well as soon as his stump is healed.

b. *An arm for appearance and work.* — This is a more complicated and esthetic contrivance than the assistance arm, but stronger and more useful. Such limbs are made up of (1) some means of suspension upon the shoulder and breast (a leather plate, a corset, a vest, supplied with straps) to which is added a leather sleeve for amputations of the fore-arm ; (2) a sheath affixed to the stump to which the professional utensils are attached, and, for the completed apparatus, the esthetic hand with an automatic thumb.

4. *For the lower limbs* the apparatus varies accordingly as the patient's stump is or is not thoroughly evolved. (This process sometimes requires 12 months.)

a. *For stumps in process of evolution* the apparatus employed is of leather with metal splints, permitting adjustment to the changing stump. Such limbs are made up of (1) some means of fixation and suspension (body belt, straps for amputations of the thigh, and a thigh band for amputations of the leg); (2) a sheath affixed to the stump, into which the weight of the body is thrown in walking; its upper edge should be modeled with great care at the points of bearing, and well padded. This sheath may later be replaced by either a wooden stump or an esthetic foot and leg, articulated at the knee freely or with a lock.

In the Belgian army these intermediary appliances are not used, the first temporary apparatus serving until the stump is fully developped.

b. *For stumps completely developed* the appliances of reinforced leather ought to be replaced by an apparatus the sheath of which is made of some rigid material (wood is used by Americans, wooden shavings glued and compressed are used by the Belgians, fibrous leather, metal plates, etc.). These appliances are much lighter, simpler, and more durable than those made of reinforced leather. Their interior surface must be exactly modelled to fit the stump and the points of support for the body when the appliance is in place. They should reproduce the form of the amputated member and consequently reestablish the normal bearing of the patient. They should also be provided with articulations which make possible movements approximating the normal.

### IX. — Fractures.

A. — Choice of apparatus.

1. In the treatment of complete fracture by war projectiles, fixation takes precedence over reduction.

2. Immobilization is necessary under three conditions; requiring different methods of procedure :

a. *Temporary* immobilization at the rescue station.

b. *Permanent* immobilization at the surgical center.

c. Immobilization *during transportation*.

Temporary immobilization ought to be as nearly as possible like the permanent immobilization, and this latter should be of a nature to permit the transportation of the patient.

Any appliance for fixation should permit the use of *mechanical extension*, *easy access to the wound*, and *the transportation of the patient*.

Fractures of the arm.

a. *Temporary immobilization at the rescue station.* — Thomas splints and crutches give excellent results. Their use should be more general in the armies.

b. *Permanent immobilization.* — The foregoing appliances, frames and adjustable brackets permitting the abduction of the member, and extension crutches, are useful in permanent immobilization.

Fracture of the fore-arm.

a. *Temporary immobilization at the rescue station.* — A grooved metal splint with an elbow at right angles, or a simple wooden splint, are sufficient.

c. *Permanent immobilization.* — The fore-arm should be immobilized with the patient in a supine position, and the appliance should allow as far as possible the application of mechanical extension and variation in the degree of elbow flexion.

Appliances of the Thomas and Sinclair type, plaster with metal splints, and the Vamelvede splint, meet the requirements.

Fractures of the thigh.

a. *Temporary immobilization at the rescue station.* — The best splint appliance is that of the Thomas type. It is well to add to the splint a foot rest to prevent the heel from being drawn forward during removal on a stretcher.

b. *Permanent immobilization.* — The appliance chosen should permit extension, abduction, and flexion of the member (splints of the Thomas type, suspensory mechanism of the Anglo-American type, the Delbet and d'Alquier appliances). The Delbet appliance permits very early walking.

The number, the seat, and the extent of lesions upon the lower limbs, and the intolerance of all appliances, may warrant the use of Finochietto's stirrup. The addition of a splint of the Thomas type to this stirrup makes possible the transportation of the patient while extension is maintained.

Fractures of the leg.

a. *Temporary immobilization at the rescue station.* — Splints of the Thomas type and grooved metal splints meet all requirements.

b. *Permanent immobilization.* — The appliance chosen should permit mechanical extension.

Extensible appliances of plaster with metal splints and adjustable posterior splint, all the extensory appliances with suspension, and the Thomas splint, meet this requirement.

It is well to begin walking as early as possible. The best apparatus for this is Delbet's, in which a lateral plastered splint is replaced by a metal splint, bridged if the position of the wound requires it.

The Finochietto stirrup, alone or with a splint of the Thomas type, meets the same requirements in fractures of the leg as in those of the thigh.

Fractures of the wrist.

a. *Temporary immobilization at the rescue station.* — A grooved metal splint or a wooden splint meets all requirements.

b. *Permanent immobilization.* — The Robert Jones splint is excellent.

Fractures of the ankle.

a. *Temporary immobilization at the rescue station.* — A grooved metal splint is sufficient.

b. *Permanent immobilization.* — Plaster appliances with bridges immobilize perfectly.

The same apparatus may be used for walking.

The metal appliance of Robert Jones will render the same services.

If in the *transportation* of the wounded the so-called permanent apparatus has to be removed, a plaster appliance may be substituted.

B. — Surgical treatment of fractures.

I. — *At the rescue station*, the treatment of fractures consists in :

*a.* The bandaging of the wound.

*b.* Immobilization.

*c.* The immediate treatment of complications (hemorrhages and shock).

Immobilization may be obtained by temporary appliances (See " Choice of apparatus ").

II. — *At the first surgical center*, all fractures should be examined, radiographed, and operated if necessary.

1. Destroyed parts and gangrenous segments should be amputated as nearly as possible *in the region of the fracture.*

2. *A seton ball wound*, with small orifices at the point of entrance and exit, and without intermediary swelling or lesions of the large blood vessels, may be immobilized in a Thomas appliance, in a grooved splint, or in plaster. The wound should be treated aseptically. The patient should be carefully observed for at least a week, and if marked local or general infection takes place, the fracture should be operated.

3. All other fractures should be operated.

a. *Treatment of the wound.* — The wound should be opened wide enough to permit a deep exploration of the entire region of the fracture. Soft parts should be excised and foreign bodies removed (according to the general recommendations for operating wounds).

b. *Treatment of the bone.* — The cutting away of bone splinters is the first essential. First remove the free splinters, those that are only slightly attached to the muscular parts. All ablation should be subperiosteal and thorough. Bony protrusions that are irregular and that endanger the nerves and blood vessels should be removed with a gouge forceps.

The cleansing solution is left to the choice of the surgeon; ether and Dakin's solution are most popular.

The wound should then be treated by *primary* or *secondary* suture.

At present primary suture of the soft parts is exceptional and is praticed only under the following conditions: the presence of an experienced surgeon; adequate supplies and a period of military calm; wounds of not more than eight hours standing; slight muscular and bony destruction, even after the excision of all the contused tissues; easy closing of the wound; and the possibility of watching the patient until the wound is completely healed. The most favorable regions are the face, the cranium, parts over flat bones, the patella, the hands, and the feet.

At the first sign of complication, the wound should be opened and cleaned.

*Secondary* suture should be preceded by progressive sterilization. The Carrel and Morison methods give good results.

III. — *Cases of fracture ought not to be moved during infection.*

IV. — *Reduction, position, and extension* of a member may be controlled by means of radiographs.

V. — Some *fractures that resist reduction* may be best subjected to internal fixation. This treatment ought to be applied so far as possible after the thorough sterilization of the wound.

VI. *Uniformity of treatment* for serious fractures at the various sanitary stations tends to give better results.

VII. — *Exercise* of the joints, muscles, and tendons of the fractured member, is requisite through the entire period of treatment and should begin as early as possible.

X. — *Treatment* of wounds of the joints.

1. At the rescue station, wounds of the joints should be immobilized with great care in some suitable appliance.

2. Except in the case of ball wounds with punctiform orifices and without fracture, which may safely wait, every wound of the joints ought to be operated upon within 6 or 8 hours at some surgical formation near the front.

With the English surgeons indication for operation consists in the extent of the wound, laceration of the articular tissues, the presence of the projectile, and, especially, fracture.

3. A radiological examination is indispensable in all cases.

4. Large aseptic arthrotomy with excision of the tract, wide exploration of the joint, careful ablation of foreign matter and bone splinters, and rectification and curetting of the bone, may be followed by complete or almost complete reunion without drainage. A compressive bandage should be applied.

5. Typical or atypical resection should be practiced only for considerable damage to the bones; at the knee it should be primary, whereas, at the elbow and shoulder, secondary resection is preferable.

6. In case of serious arthritis with suppuration, first try large arthrotomy with complete immobilization and progressive disinfection of the wound. In case this treatment fails, resection followed at once by separation of the bony surfaces should be practiced. In very serious cases immediate resection is admissible.

7. If there is considerable damage of the articulating bones together with epiphysial fracture of both segments, accompanied by serious infection, recourse must be had to amputation.

After a simple operation M. Gosset prefers a padded compressive bandage without immobilization.

MM. Depage and Tuffier insist that the limb should be completely immobilized for 8 or 10 days, after which time mobilization may begin.

MM. Willens and Derache believe that mobilization should begin much earlier.

### XI. — Wounds of the blood vessels.

A projectile in the neighborhood of a blood vessel may cause either contusion or a wound (incomplete section, complete section, or perforation).

Contusion is evidenced by thrombosis which is usually unrecognizable until embolism or a secondary hemorrhage takes place.

Wounds of the blood vessels betray themselves generally by an immediate external hemorrhage in the case of large wounds or by the presence of diffuse hematoma in the case of narrow tracts.

After patients have been removed to the hospital of the district, arterial or arterio-venous traumatic aneurysms resulting from a vascular wound which have escaped attention, may be detected.

Treatment. — 1. Contusion should be treated by ligature below and above the thrombosis. Arteriotomy followed by suture must be rejected.

2. Vascular wounds with external hemorrhage call for treatment which begins at the rescue station and is completed at the hospital.

a. *At the rescue station* the treatment should be varied according to the amount of the hemorrhage. If the flow is slight, compression above the wound or in the wound itself may be produced by bandages. If the hemorrhage is serious, a tourniquet must be applied. *All cases with tourniquets should be clearly designated and removed immediately to a surgical station for operation.* Our English colleagues, because of the peculiar conditions attending their removal of the wounded, employ forcipressure and ligature instead of the tourniquet.

b. *At the surgical station*, as a rule, the injured vessel should be laid open and *ligatures* applied to the healthy parts of the two ends. Vascular suture, whether lateral or circular, is the ideal operation if conditions are favorable (that is, when the vascular lesions are limited and easily accessible and the wounds aseptic and capable of being completely closed).

3. *Secondary* arterial hemorrhages should be treated by ligature of the two ends of the injured vessel, or, if this treatment is impossible, by the application of fixed forceps or by ligature at a distance: amputation is the last resort.

4. In vascular lesions with diffuse hematoma *preventive hemostasis* must first be effected (tourniquet, compression of the artery after exposure from above); then a wide opening of the hematoma; finally ligatures should be applied to the ends of the injured vessel, or, as is seldom possible, suture may be effected. In case the lesion is so situated as to endanger the reestablishment of the circulation (popliteal, common femoral, axillary) the temporary introduction of a paraffin tube bound to the two ends of the vessel, diminishes the danger of gangrene.

Diffuse hematoma is often complicated with gangrene. Ligatu[illegible] of the vein at the same time as ligature of the artery instead of increasing the risk of gangrene, as one might suppose, actually diminishes it. Facts tend to prove that in the case of lesion of the arteries, simultaneous ligature of the uninjured vein is desirable.

5. In *traumatic aneurysm*, immediately after hemostasis has been effected, the sack should be opened; in this way access is obtained to the primary vascular lesion, and ligature may be applied close to the lesion thus avoiding injury or exclusion of the collateral branches in the near neighborhood.

*a*. In *arterial aneurysm*, the ligature of the two ends should be followed by the complete removal of the sack, or the partial removal of it in case complete removal is dangerous. Suture of the arterial orifice is here again the ideal operation if it can be accomplished.

*b*. In *arterio-venous aneurysm*, more common and less dangerous than arterial aneurysm and less frequently complicated with gangrene, the ideal operation is the removal of the sack and the reestablishment of arterial and venous continuity by means of suture. In practice, ligature of the four ends followed by the total or partial removal of the pocket is the usual procedure.

### XII. — Wounds of the nerves.

1. In surgical formations at the front it is necessary to search with utmost care for lesions of the peripheral nerves by clinical examinations and examinations made directly in the wound at the time of the first treatment.

2. Whenever a peripheral nerve trunk is found to be severed, primitive suture of the nerve should be effected if the conditions of the wound permit, and mention of this suture should be made on a special bulletin accompanying the patient.

3. If the nerve could not be sutured at the time of the first treatment, or if it was overlooked, the necessary operation may be performed at the time of the union by second intention.

4. This operation, whether primary or secondary, may of itself suffice for the restoration of the nerve function. In any case it prepares the nerve trunk in the best way possible for subsequent treatment if such proves necessary.

5. Later on, all functional defects resulting from nerve lesions may be subjected to operations in accordance with the advice of a neurologist.

6. During the whole course of the treatment a favorable position of the segments of the member should be maintained, and the play of the joints and nutrition of the muscles carefully attended to.

7. *Operations* upon the nerves, whatever their character (liberation or suture), demand perfect asepsis, much attention to detail, and extreme gentleness in all action upon the nerve itself.

8. The *suture* should be made upon refreshed nerve extremities extending on to the healthy tissue : a lower end of normal appearance and freed from all fibrous tissue which might constitute a hinderance to regeneration, should be utilized.

9. Contact between two nerve ends appears necessary to regeneration; nevertheless suture at a distance, and splitting of the lower end may be tried when other treatment is impossible.

10. So far graft has produced no definite results.

11. Instead of nerve suture, recourse may be had to tendon transplantation : (1) when suture of the nerve is impossible; (2) when the functional result, in spite of the operation upon the nerve trunk, is unsatisfactory.

### XIII. — Wounds of the spinal cord.

1. *At the rescue station*, practice the usual bandaging and deal with the shock phenomena which frequently attend wounds of the spinal cord.

2. *At the surgical formation* :

*a.* If there is a ball wound with a punctiform orifice, bandage and observe.

*b.* If there is a shell wound with a large entrance orifice, treat the wound as usual without paying attention to the medullar lesion.

3. Remove the patient at the earliest opportunity in a Bonnet grooved splint.

4. From a therapeutic point of view there are two altogether distinct classes of wounds of the cord.

*a.* Complete section, against which surgery is powerless.

*b.* Compression by a projectile, by splinters, by the displacement of bones, or by meningeal hemorrhage.

Intolerable pain may be a decisive indication for treatment.

5. When is it best to operate?

In most cases primary treatment is out of the question. In the Italian army however operation as early as possible has been decided upon for bone compression and the entrance of projectiles into the spinal cavity, so as to remove the projectiles, bits of clothing, clots, and splinters, and thus free the cord and the spinal veins. The mortality resulting from these primary operations is very high. In order to reduce as much as possible the traumatism of the operation and to conserve the spinal column, *unilateral* laminectomy has been found preferable in the Italian army.

6. The secondary operation should take place after the healing of the wound.

With a view to remote prognosis, an absolute distinction should be drawn between lesions at the level of the cord itself and those on the level of the cauda equina.

### XIV. — Cerebral wounds.

1. At the rescue station cerebral wounds should be covered with a simple aseptic dressing.

2. If there is any considerable loss of tissue or serious complications, cases of cranial and cerebral wounds should not be transported for any great distance.

If the lesion is not so serious and if there is no grave menace from complications, the patient may be removed to a hospital center, provided with a special service of surgeon and neurologist, where he may remain for a considerable time.

Operation should be early.

3. A case of cerebral wound ought not to be transported within three weeks after trephining for cranio-cerebral lesion.

4. The cranium should be carefully examined by X-ray and the projectiles and bony fragments exactly noted.

5. Indications for trephining are still under discussion in cases in which the fracture of the cranium is not evident, and when there is no cerebral symptom.

6. Local anesthesia is preferred for the operation. The sitting posture tends to diminish hemorrhage, and is easily maintained in secondary or delayed operations.

7. The cleaning of the wound is made by resection of the edges. Enlargement is practiced according to the seat, the direction, and the form of the lesions of the bone.

8. Bone should be removed to just beyond the limit of cerebral contusion. This is preferably done by means of gouge forceps, exceptionally with trephine, but never with chisel and mallet.

9. If the dura mater is intact, it should not be disturbed; and in favorable cases the wound may be closed by first intention. If it is torn, its edges should be rectified and the opening increased to the limits of the cerebral lesions.

10. Clots, diffluent cerebral matter, and foreign substances on the surface should be removed by ablation with a warm saline solution; deep splinters and easily accessible projectiles should be removed with the greatest precaution.

11. Small projectiles that are deeply imbedded and difficult of access, larger projectiles at the base of the cranium or in the ventricles, and those in the hemisphere opposite to the one with the lesion, should not be disturbed.

12. In bipolar wounds, each opening should be treated separately, and the deep seated part of the tract should be left undisturbed.

13. No drain or gauze should be placed in the cerebral substance. A cranio-cerebral wound may be treated by primary or secondary suture after sterilization. Research work is still desirable to determine which of these two methods is the more effective means of avoiding immediate, secondary, or late infection.

14. After the operation, the patient should be given a half sitting posture.

15. Early cerebral hernia without deep infection should be treated by enlarging the bone breach.

16. Small scars with large loss of bone, and those which give rise to cerebral troubles, should be treated by cranioplasty.

17. Complications should be treated, according to their origin (cerebral abcess, foreign matter, adherent scars), by suitable operations to be determined by a neurologist and surgeon in consultation.

### XV. — Wounds penetrating the chest.

1. Wounds penetrating the chest result in a mortality estimated at 20 o/o in the sanitary formations of the army zone.

2. Early death is the rule because of asphyxiation and hemorrhage. Shock plays a large part in the early or immediate death.

3. Late mortality usually results from pleuro-pulmonary infection.

4. Pathological anatomy establishes that the pulmonary lesions are the same as those of all war wounds. The mechanical disturbances are the same, the infection is the same, — except that pulmonary tissue resists infection better than other tissues, — but the infection of the pleura either by the external air or by the pulmonary wound, may seriously complicate the case.

5. Two points in the treatment are of utmost importance : immediate attention and complete immobilization of the patient.

When the patient is suffering from shock, general treatment for shock should be undertaken and the patient should be given a reclining posture with the head lowered.

*Medical* treatment is sufficient for the healing of many chest wounds wich present no complication.

*Surgical* treatment should be employed for parietal lesions and for immediate or late complications.

In all cases complete surgical treatment of thoracic lesions

(wounds of the soft parts, fractures of the ribs, etc.) is absolutely necessary, as in all war wounds.

A. *Immediate complications.*

a. *Opening of the thorax.* — Closing is necessary either by plugging or by direct suture of the wall.

b. *Hemorrhage.* — *With open thorax* : Direct hemostasis of the lung either by plugging or by hemostatic suture. *With closed thorax* : If it is certain that shock is not the primary cause, and if the blood pressure drops in spite of treatment, and if there are symptoms due only to hemorrhage, the ideal operation is thoracotomy with direct hemostasis of the lung. Indication for operation in cases of closed thorax is very rare. Such treatment requires competence and equipment of the highest order.

c. *Hemothorax.* — If hemothorax is discovered, puncture to relieve mechanical compression is permissible. It is well during or after the drawing of the blood, to inject air or oxygen into the pleura, in order to prevent hemostatic collapse of the lung. Febrile hemothorax calls for exploration by repeated punctures for the bacteriological examination of the liquid.

B. *Late complications.*

a. *Persistent aseptic hemothorax.* — This variety of hemothorax should be treated by repeated puncture for evacuation to allow of pulmonary expansion. The introduction of a certain amount of oxygen into the pleura, during or after these punctures, is often useful.

b. *Infected hemothorax.* — When infection of the pleural fluid has been established by bacteriological examinations, thoracotomy is definitely indicated.

c. *Purulent pleurisy.* — This may be treated in the same way as infected hemothorax. When drainage is deemed necessary, it should always be effected at the lowest point (the posterior base of the thorax).

6. Treatment of pleural suppuration by the method of progressive sterilization.

In recent or old cases of pleural suppuration there may be practiced progressive sterilization and secondary suture of the thoracic wall. There is no reason to fear a suture of the thoracic wall over an empty cavity. It is the best treatment to obtain rapid expansion of the lung and the disappearance of the pleural cavity (Depage).

7. In urgent cases, it is well to proceed at once to the extraction of intra-pulmonary projectiles when conditions are favorable.

8. Whatever the circumstances, prophylactic therapeutics of the pleuro-pulmonary infection by means of direct surgical treatment of the lung wound (extraction of all foreign bodies, suture of the wound with or without excision) seems the logical procedure. This question deserves careful investigation.

9. Blood effusion in the pericardium is governed by the same therapeutic principles as hemothorax.

### XVI. — Abdominal wounds.

1. On general principles, all recent wounds of the abdomen should be operated, except when the lesion is without doubt limited to the liver or the kidney, and when there is no symptom of serious hemorrhage.

2. The operation should be performed as early as possible, except when the patient is suffering from shock. When there is doubt as to the relative seriousness of the shock and that of the hemorrhage, it is better to operate. Do not make a practice of laparotomy later than 36 hours.

3. Centers should be established as near to the lines as possible, provided *with all surgical appliances which are now recognized as necessary*, where all cases of abdominal wounds may be taken for operation by competent surgeons.

